# Refugees in Canada during the First Wave of the COVID-19 Pandemic

**DOI:** 10.3390/ijerph18030947

**Published:** 2021-01-22

**Authors:** Jennifer Edmonds, Antoine Flahault

**Affiliations:** Institute of Global Health, Faculty of Medicine, Université de Genève, 1211 Geneva, Switzerland; Jennifer.Edmonds@etu.unige.ch

**Keywords:** COVID-19, refugees, Canada, border closures, economic measures, health literacy, access to healthcare, health insurance

## Abstract

It is crucial to understand how the most vulnerable populations have been impacted by the ongoing COVID-19 pandemic. This paper intends to contextualize the experience of resettled refugees in Canada during the COVID-19 pandemic, framing the issue for further study as the situation evolves. Based on the experience drawn from the first wave of the pandemic, the findings of this paper suggest that refugees in Canada encounter barriers to healthcare, economic support, education, social support, and border crossing impediments, all of which can have a compounding effect. These findings provide needed information to inform the development of effective policies and strategies to support refugees during health security emergencies in Canada.

## 1. Introduction

In December 2019, a new coronavirus emerged in China. COVID-19, the novel infectious disease caused by the SARS-CoV-2 virus has proceeded to threaten and overwhelm public health systems around the globe. As of 6 May 2020, there have been over 3.7 million cases worldwide and over 259,000 deaths [1], with an infection fatality rate estimated to be between 0.5% and 1% (Antoine Flahault, personal communication, from the serosurveys conducted in Geneva). On 11 March 2020, the World Health Organization (WHO) declared the outbreak of COVID-19 a pandemic, at which point Canada had publicly reported 103 cases [2]. As of 6 May 2020, this number had jumped to 63,281 known cases of COVID-19 in Canada [1].

As countries develop policies and programs to manage COVID-19, health security has been brought to the forefront of national and international governance. Health security has been acknowledged to be one of the most important non-traditional security issues at both national and international levels, a point which has been further highlighted in the context of a pandemic [3]. The term encompasses two differing approaches to protecting health from threats: individual health security and collective health security. Individual health security refers to “security that comes from access to safe and effective health services, products, and technologies” [3] (p. 1884). Conversely, collective health security is defined as “reducing the vulnerabilities of societies to infectious disease threats that spread across national borders” [3] (p. 1884). Both approaches are pertinent to the fight against COVID-19 as countries scramble to both contain the spread of the communicable disease and work to strengthen their health systems to manage the influx of patients.

The health security measures enacted by authorities at various levels and orders of public health and government have significantly affected how society operates, at both the individual and population level. While the majority of people will be affected by the pandemic in some form or another, it is necessary to consider the experience of the most vulnerable in our society. As discussed by Christine Straehle in Ethical Reflections on Who is At Risk: Vulnerability and Global Public Health, “to be vulnerable means to be unable to protect one’s fundamental interests effectively” [4] (p. 196). Straehle goes further to point out that there are some whose very circumstances make them more open to harm, affecting their ability to protect their fundamental interests. It is this notion of circumstantial vulnerability that is fundamental to the aims of this paper, focusing on the experience of refugees in Canada [4].

As of June 2019, there were over 25.9 million refugees globally, a number that is projected to grow [5]. While the majority of refugees are hosted in low- and middle-income countries, resettlement efforts in high-income countries have greatly increased in the past few years [6]. For instance, in 2018, 92,400 refugees were resettled in 25 countries, with Canada admitting the most (28,100) [7]. As one of the top destinations for refugee resettlement in the world, it is necessary to examine the experience of refugees in Canada during this pandemic. This paper will demonstrate that refugees are a circumstantially vulnerable group, aiming to explore how refugees resettled in Canada are impacted by the health security measures enacted to manage the COVID-19 pandemic.

This paper endeavours to frame the issue for future research, but not to assess the impact of the health security measures themselves, or to evaluate which methods were more or less effective than others in the effort to combat COVID-19. As COVID-19 is an evolving situation, this paper aims to outline the importance of considering the experience of the refugee in Canada during the COVID-19 pandemic. A deeper analysis of the impact of the health security measures enacted is an area for further research.

## 2. Materials and Methods

Looking at Canada’s experience as a case study, a scoping review of peer-reviewed and grey literature related to the challenges facing resettled refugees during the first-wave of the pandemic was conducted. Refugees are defined as those “who [are] unable or unwilling to return to their country of origin owing to a well-founded fear of being persecuted for reasons of race, religion, nationality, membership of a particular social group, or political opinion” [8] (p. 3). Aiming to provide a holistic understanding of the issue, scholarship from different disciplines was used throughout the manuscript, including education, international relations, economics, healthcare, religion, and sociology.

### 2.1. Search Strategy

The literature was retrieved from a structured search of biomedical databases and the Web.

**Figure 1:**
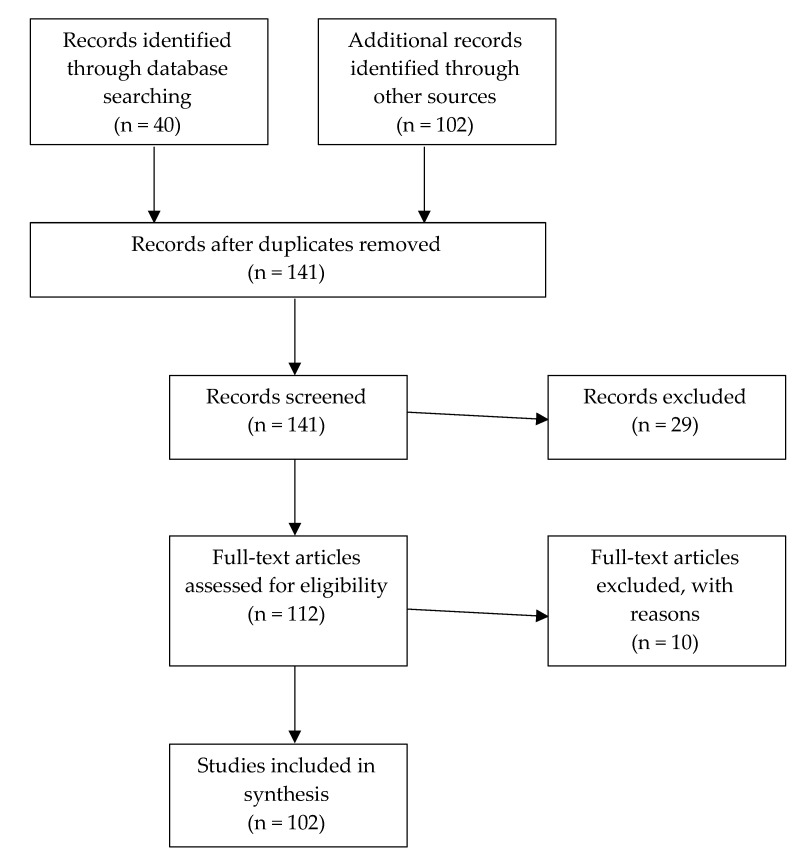
Process of selection of reviewed records.

An initial search of biomedical databases (PubMed and JSTOR) focusing on the articles’ titles was conducted. The following keywords had to be present in the title: “COVID-19; refugees.” Articles had to be written in English or French. We searched for articles, reports or editorials, published between January 2020 to May 2020. The scope was then broadened through a second search, including all fields in the search (search methods selection process is summarized in Figure 1). The last search was conducted on 15 May 2020. This search revealed scant literature, in large part due to the newness of the virus.

In order to find potentially additional relevant sources, a screening and identification of primary sources and publications from academic journals outside the aforementioned timeline was undertaken. The Government of Canada was a prominent source for contextual information regarding the Canadian refugee process.

### 2.2. Exclusion Criteria

Exclusion criteria was developed to identify literature relevant for consideration within the review. Literature was excluded that focused on refugee camps rather than resettled refugees. As well, it was not included if it did not comply with the geographic area or definition of refugees relevant to this paper.

### 2.3. Data Extraction

For data extraction, an excel spreadsheet was used to complete the following information: Title, Author(s), Date of publication, Study location focus, Type of document, Main topic, Population.

## 3. Results

### 3.1. Brief Overview of the Refugee Resettlement Programs in Canada

Refugees resettling in Canada are either government sponsored, privately sponsored, or part of the Blended Visa Office-Referred (BVOR) program. In the Government-Assisted Refugees (GAR) program, resettlement support is entirely the responsibility of the Government of Canada or the Province of Quebec. Immigration, Refugees and Citizenship Canada (IRCC) provides funding to non-government agencies who deliver this support [9]. In contrast, Privately Sponsored Refugees (PSR) are funded by a group of people in Canada who volunteer “to give emotional and income support to the refugees for the full sponsorship period” [10]. Since its inception 40 years ago, the PSR program has supported over 327,000 refugees resettling in Canada [11]. The sponsorship period for both the GAR and PSR programs is usually one year. A union of the GAR and PSR programs, the BVOR program, involves support from both the government and private sponsors, who each supply 6 months of financial support. As well, private sponsors must provide a full year of social and emotional support [12]. Generally, privately sponsored refugees have better earning and employment outcomes than those sponsored by the government because of the integrative nature of the program [13]. The Canadian government creates an annual target for each category. The 2019–2021 Immigration Levels Plan currently aims to intake 51,700 refugees and protected persons in 2021: 10,700 GARs, 20,000 PSRs, 1000 BVORs, and 20,000 protected persons in Canada and dependents abroad [14].

The In-Canada Asylum Program allows asylum seekers in Canada to make a refugee claim at a port of entry or at an in-land office [15]. However, in 2004, an agreement between the United States and Canada, the Safe Third Party Agreement, came into effect, requiring refugee claimants “to request refugee protection in the first safe country they arrive in” [16]. Accordingly, asylum seekers attempting to enter Canada via a port of entry along the Canada-United States border do not meet the eligibility requirements to file a refugee claim and will be returned to the United States. The Safe Third Party Agreement has been met with controversy in recent years, as refugee advocates denounce the claim that the United States is a safe country for all refugees [17].

### 3.2. Access to Healthcare

Diseases do not affect everyone equally, and COVID-19 is no different. Infectious diseases often impact marginalized populations disproportionately, highlighting inequities in access to care and the importance of social determinants of health. Consider TB, a curable disease which kills millions annually. This disease largely affects marginalized populations, such as the socially and/or economically excluded, particularly thriving in conditions of poverty [18]. In Canada, these health inequities are often highlighted when the health of First Nations, Inuit, and Metis is compared against that of the general population. For example, in 2014, the Inuit population had a rate of new or retreatment cases of TB that was almost 50 times higher than that of the overall Canadian population [19]. Vulnerable groups are often hit hardest by disease, a pattern further underscored during the COVID-19 pandemic.

Individual health security measures ensuring access to a strong health care system for the entire population are critical for success in public health, especially within the context of a pandemic. The provision of quality healthcare for all provides a level of security for all, not just the ones in need. If everyone has access to evidence-based information, diagnostic tests and care, the SARS-CoV-2 virus will be less able to transmit freely throughout the population and infect others. This is particularly important for resettled refugees who are often managing a variety of acute and chronic diseases such as tuberculosis (TB), malaria, hepatitis, intestinal parasites, nutritional deficiencies, diabetes, and hypertension [20,21]. Knowing that comorbidities such as cardiovascular disease, obesity, and diabetes are major risk factors for COVID-19, ensuring access to healthcare services among high-risk groups such as refugees is key to effectively managing the pandemic [22]. Singapore, which had previously been lauded for its handling of the pandemic, has recently seen an increase in cases from migrant workers. The Ministry of Health reported on 6 May 2020 that 88% of the cases nationwide were in migrant worker dormitories, thereby illustrating the importance of ensuring healthcare access for the most vulnerable [23]. This need has been highlighted in Canada by the preliminary data released on 5 May 2020, indicating that areas with higher numbers of recent immigrants (including refugee groups) and low-income earners in Toronto, Ontario, have experienced higher rates of COVID-19 infections and hospitalizations [24]. In Toronto, the low-income earning quintile had 113 cases per 100,000 people, compared to 73 cases per 100,000 people in the highest-income quintile. Applying the same process to newcomer data, Toronto Public Health found similar results indicating being a newcomer to Canada was a risk factor: areas with more recent immigrants had more COVID-19 cases (104 cases per 100,000 people) while areas with less recent immigrants had less COVID-19 cases (69 per 100,000 people) [25]. As a disproportionately impacted group, it is essential that the equity in access to health care for newcomers, including refugees, is examined.

### 3.3. Health Insurance

The modern Canadian healthcare system was founded on the principle of providing care on the basis of need rather than ability to pay [26]. It is a publicly funded decentralized universal health care system, with provincial and territorial health insurance plans covering the population. As a result, most healthcare is free at the point of care. However, non-citizens must apply for coverage from the province or territory in which they reside, a process which takes a minimum of three months in Ontario, Quebec, and British Columbia (where 80% of Canada’s newcomers arrive), and sometimes as long as two years [27].

To bridge the gap between arrival in Canada and qualifying for provincial or territorial health coverage, the federal government offers the Interim Federal Health Program (IFHP). The IFHP provides “limited, temporary coverage of health-care benefits for specific groups of people [including resettled refugees and refugee claimants]” [28]. This coverage is extended until the individual qualifies for provincial or territorial health insurance or, in the instance of rejected refugee claimants, their access to basic coverage continues until they leave the country [29]. Therefore, at the policy level, refugees always have access to healthcare, a necessary step to ensure they can achieve good health status. However, in practice, the healthcare needs of refugees are not always being met. Research exploring the healthcare needs and use of the healthcare system by Syrian refugees resettled in the Greater Toronto Area, in Ontario, found that almost half (49.0%) of the study’s participants reported unmet healthcare needs. In comparison, only 11% of the general Canadian population has reported the same unmet healthcare need [30].

There are many barriers inhibiting the achievement of individual health security among the resettled refugee population in Canada. For instance, refugees must apply for IFHP coverage, requiring them to overcome the administrative hurdles inherent in this process, including finding the correct documentation and overcoming language barriers to complete the requisite forms. This barrier echoes the results of a study performed in San Diego, California, which found language barriers to be a major impediment negatively affecting resettled refugees’ ability to access good healthcare [20]. As well, a study conducted in Hamilton, Ontario, found that the IFHP was unknown to most healthcare providers and clients [refugees]. Moreover, it was misunderstood by most of those who were aware of it because of the many complexities within the system, thereby indicating the dearth of knowledge regarding healthcare resources for refugees amongst those who could most benefit from it [31].

Recognizing the importance of everyone having access to COVID-19 healthcare services, certain provinces, like Ontario, have lifted the usual three-month wait to access their health insurance plans. Anyone experiencing COVID-19 symptoms, including refugees who have not yet qualified for the Ontario Health Insurance Plan (OHIP) coverage, could access testing and treatment free of charge as of 19 March 2020 [32]. However, this presents similar obstacles to the IFHP, as individuals who could benefit from this measure first need to know about it, a challenge when the individual is not familiar with the healthcare system or does not have the means to access this information. As well, while OHIP coverage will start immediately for refugees, they still need to apply for it, which involves the same bureaucratic challenges as the IFHP. The potential language barriers or a poor literacy level further compounds this challenge [33].

### 3.4. Health Literacy

Misinformation has been a major challenge in the fight against COVID-19, prompting the WHO Director-General Tedros Adhanom Ghebreyesus to say “we’re not just fighting an epidemic; we’re fighting an infodemic” [34]. Conspiracy theories and unproven cures touted as miracle treatments, some actually harmful, have circulated on social media at an extremely fast pace. Rumours have covered everything from the origin and cause of the virus, symptoms and modes of transmission, prophylactics and treatment, to the success of various health interventions [34]. For example, gargling colloidal silver has been colloquially promoted as a way to strengthen one’s immune system against COVID-19, despite the lack of evidence proving the effectiveness or safety of the marketed prophylactic [35].

The spread of this false information can have devastating consequences. Strong health literacy is therefore an important skill to facilitate the navigation of COVID-19 misinformation. Canada’s Public Health Association has defined health literacy as “the ability to access, understand, evaluate and communicate information as a way to promote, maintain and improve health in a variety of settings across the life-course” [36] (p. 11). Health literacy is thus crucial for the attainment of good health status, affecting people’s ability to navigate fact from fiction in the COVID-19 pandemic.

Understanding the health literacy levels of refugees in Canada is therefore necessary in order to predict and comprehend how they will be impacted by the infodemic and consequently the pandemic. However, there is a lack of research exploring the health literacy levels of refugees, though some literature examining that of immigrants is enlightening. In 2010, Ng and Omariba et al. studied the connection between health literacy and immigration in Canada, developing a Health Activities Literacy Scale to score participants’ level of health literacy. The researchers determined that participants needed to score a minimum of 275 points to demonstrate the level of health literacy needed to maintain their health. Their findings revealed that while 45% of non-immigrants scored the minimum 275 points, only 25% of immigrants attained this score [37]. Though Ng and Omariba’s study looked at immigrants, not refugees, the results nonetheless illuminate a disparity in health literacy amongst newcomers to Canada. Accordingly, these individuals are more susceptible to the false information circulating about COVID-19, potentially resulting in unnecessary health and/or financial loss.

Issues of trust further compound the challenges raised by poor health literacy levels, affecting the absorption of needed information related to COVID-19. As outlined by Tricia Hynes, relationships of mistrust are prevalent in all eight stages of the refugee experience (“the period of threat; the decision to flee; in flight; reaching safety and a place of asylum; the refugee camp experience; reception into a host country; resettlement; and post-resettlement”), which contribute to issues of trust [38] (p. 1). The fact that the feelings of mistrust in the last three phases are often directed towards the host government and population is of particular significance to the context of COVID-19 [38]. At a time when misinformation is spreading quickly via social media and other avenues like WhatsApp, mistrust directed at the government could turn refugees away from reputable knowledge sources. Individuals may turn towards peer-to-peer sources of information, which are more likely to carry false information. Dr. Rhoopali Chaudhary, founder of Lotus STEMM [39], an Ontario-based initiative translating COVID-19 information into South Asian languages, addressed this challenge:

“*We don’t have debunking happening in the native language that people are speaking so people are more inclined to believe things that’s coming through WhatsApp or Facebook because it’s either coming from someone that they trust, or it could just be family and friends back home sending it in a language that they emotionally connect with*.”

[40]

As well, social distancing has made people heavily reliant upon social media, using it to get and spread information about COVID-19 [41]. Though WhatsApp has now taken measures to limit the forwarding capability in the application, the Facebook owned company has acknowledged that misinformation has been spreading on the platform [42]. It is important to note that not all refugees share these issues of trust, nor are refugees unique in their susceptibility to misinformation. Instead, this article endeavours to point out the various factors which contribute to the challenges inhibiting refugees’ good health status amidst the COVID-19 pandemic, including low levels of health literacy and issues of trust.

### 3.5. Personal Protective Equipment and Testing Capacity

Central to achieving individual health security as part of the response to COVID-19 is strong diagnostic testing capabilities and access to personal protective equipment (PPE) available for all Canadians, including refugees. This has been a source of difficulty for Canada, where both have been in short supply as demand across the world has skyrocketed [43]. COVID-19 testing capacities vary by province. As of 1 May 2020, Alberta and the Northwest Territories led the country in the rate of daily tests performed (114 and 100 daily tests performed per 100,000 people). Conversely, Quebec and the Yukon trailed behind the rest of the provinces, each performing only 29 and 27 tests per 100,000 population [44]. Dr. Theresa Tam, the Chief Public Health Officer of Canada, has said that testing capacity needs to increase to reach 60,000 tests per day [45]. As of 27 April 2020, the total number of tests performed was 26,000, indicating a need to further increase capacity [45].

PPE shortages have been a major concern for the medical community, being a crucial means to protect healthcare workers from infection and consequently stop the virus from spreading to other patients. For the general public, guidance directing face mask use has shifted as the situation has evolved in Canada. Dr. Tam has reversed earlier guidelines and now suggests the use of a non-medical mask in addition to practicing social distancing and hand hygiene to stop the spread of the virus [46]. Many homemade mask how-to guides have cropped up online, including both sew and no-sew options, making masks accessible to all, assuming individuals have internet access and the literacy skills to consume the information [47].

### 3.6. Economic Measures

Social determinants of health such as education, gender, ethnicity, and income, influence people’s health, with income potentially having the greatest impact [48,49]. Income directly shapes other major health determinants such as housing or diet. Income also affects one’s psychological well-being, and health-related behaviours such as alcohol abuse, tobacco use, and physical activity. As income rises, so do health outcomes, just as health outcomes decline as income lowers [49]. Consequently, the potential income and employment losses stemming from the pandemic will likely further compound the challenges resettled refugees’ face in attaining good health status in Canada.

COVID-19 has devastated economies around the world, with data collected by the C.D. Howe Institute Business Cycle Council indicating that the Canadian economy entered into a recession in the first quarter of 2020 [50]. The weakness in the economy will have a direct impact on individuals in Canada, with both short- and long-term consequences. Already, the Conference Board of Canada has found that the number of job postings dropped by 50% from the beginning to the end of March 2020, continuing to decline in April 2020 [51]. Moreover, in March 2020, the unemployment rate in Canada increased to 7.80%, a 2.20% increase from February 2020 [52].

As the situation evolves, the longevity of COVID-19′s impact on the Canadian economy will become clearer. Nonetheless, the results from a study conducted by Joshua Mask investigating the impact for refugees immigrating to the United States during a recession are instructive, finding that “for every one percentage point increase in the national unemployment rate at arrival, refugees on average experience a 2.99% reduction in wages five years later and a 1.8 percentage point reduction in employment four years later” [53] (p. 1). An area for further research within the Canadian COVID-19 context, the results of Mask’s study indicate that refugees may be particularly impacted by the present economic downturn.

Acknowledging the economic hardship people in Canada are experiencing because of COVID-19, the Government of Canada has implemented various economic measures meant to ease financial stress. The biggest and most applicable of these new financial supports is the Canada Emergency Response Benefit (CERB), a taxable benefit of $500 per week that qualifying individuals can apply to for up to four months [54]. In order to qualify, applicants must have stopped working or had their workload reduced for reasons related to COVID-19, thereby having an income of less than $1000 (CAD) per month. As well, applicants had to have earned a minimum of $5000 (CAD) in 2019 or in the last 12 months, and they must be a Canadian citizen or a permanent resident [55]. If an applicant has temporary status in Canada, they can only apply if they are legally allowed to work [56]. The application process is quick and easy, with options to apply online or in-person, and applicants will receive the payment within ten business days, either via direct deposit or a cheque [57]. Applications for the CERB opened on 6 April 2020, and by 13 April 2020, nearly 3.5 million people in Canada had applied [58]. Refugees, refugee claimants, and failed claimants in the midst of appealing the decision are eligible to apply for the CERB assuming they meet the requirements. However, as refugees have the lowest employment rates among all categories of immigrants in Canada [59], meeting the $5000 (CAD) income requirement in 2019 potentially excludes many of them.

Currently, everyone who applies for the CERB receives the benefit, regardless of whether or not they qualify, meaning some people who are not entitled to the funds are still getting them. Employees at the Canada Revenue Agency (CRA) have stated that though the vast majority of applicants are eligible, it is likely that many are not, though at this stage it is unknown how many CERB recipients are not entitled to the funds [60]. The CRA has stated that they will be back-checking eligibility and those found ineligible will be required to pay back the funds received [55]. Malicious intentions should not be assumed, as eligibility requirements have shifted since the initial rollout leading to confusion and many applicants unsure of their eligibility were pressured by friends or family to apply [60]. Language and cultural barriers, poor literacy levels, and unfamiliarity with Canada’s financial and tax systems challenge refugees’ ability to successfully navigate the economic supports instituted to support people in Canada during the pandemic [61].

For any refugees who may have mistakenly applied, the consequences could be serious. Consider the 2016 income of Syrian refugees who came to Canada in November and December 2015: GARs averaged $20,000 (CAD) and PSRs averaged $15,600 (CAD). The average 2016 income of GARs coming from other countries was $17,700 (CAD), and $18,200 (CAD) for PSRs. These incomes include government transfers, earnings (wages and self-employment income), and other sources such as investment income [62]. With incomes as low as these, if refugees spent the CERB when they did not in fact qualify for it, it is unlikely they will have the savings to pay back the benefit, which could be as much as $8000 (CAD), which is almost half of the income refugees reported in 2016. GARs already have a significant burden of debt as they are expected to pay the Canadian Government back for their pre-departure medical exam and their transportation to Canada, a loan which charges interest [63]. Misunderstandings related to the CERB could therefore increase refugees’ debt by thousands of dollars. The additional income could also threaten refugees’ eligibility for certain tax credits [60].

### 3.7. Closure of Public Spaces: School

Intending to limit the spread of COVID-19, all schools across the country were closed in mid-March [64]. Though the transition to distance learning has no doubt been challenging for Canadian families, it has particular ramifications for resettled refugees in Canada. While refugees are enrolled in the Canadian education system soon after arrival, many of them are coming from refugee camps (where a stay of 5–10 years is not uncommon) [65] or directly from war-affected countries, where access to quality education is both uncertain and fraught with challenges [66]. Refugees are thus already working to overcome learning gaps resulting from interrupted access to education [67]. Unfortunately, the shift to distance learning will likely further compound the difficulty refugees face in catching up to their peers’ in academia, as expounded on below.

Education is recognized as an empowerment right because it “is the primary vehicle by which economically and socially marginalized adults and children can lift themselves out of poverty and obtain the means to participate fully in their communities” [68]. However, equity of access has been a concern for many when education was shifted outside the classroom. Consider, for example, the barriers associated with virtual learning, which requires students to have access to high-speed internet and computers in order to participate.

Distance learning and virtual learning are not synonymous, and school boards such as the Peel District School Board in Ontario have stated that distance learning should “not always require online access” [69] (p. 3). In practice, however, virtual learning has been a key pillar in the implementation of distance learning, as seen in Peel’s Scaffolded Implementation Plan for Educators [69]. Nonetheless, school boards have been cognizant of the barriers implicit in a virtual learning approach. For example, technology surveys were extended to students to determine the extent of the technology needs within school boards, the results of which prompted the Toronto District School Board to distribute 28,000 laptops and iPads (some of which had built in Wi-Fi) to students found in need [70]. The results of the technology surveys have not yet been published; therefore, at this point, it is unknown whether the distribution of technology resolved the need.

Beyond the capital and technological resources required to engage in virtual learning, a degree of fluency in the language of instruction is needed for both virtual and distance learning. Earlier studies have found that it can take refugee children four to five years to acquire the level of proficiency in the language of instruction needed to succeed [65]. Moreover, parents are needed to fill certain gaps for at home learning, with the time commitment varying according to the student’s age and the approach taken by their teacher [71]. Consequently, language presents a barrier for many refugees and other immigrant families, as these parents will struggle to support their children’s education at home, an already daunting task for many. Ministries of Education across the country have acknowledged the special needs of English Language Learning (ELL) students and have asked schools to develop plans to meet these needs within their overall approach to distance learning, but little has been done to aid teachers in their efforts to support these students [71].

Refugee students’ ability to cope with and manage the format changes in education will vary based on contextual and individual factors. These factors include country of origin, ethnicity, culture, religion, socio-economic and educational background prior to resettlement, age at the time of flight, migration and resettlement, personality characteristics, and the level of family support [65]. Nonetheless, education is considered “a means of creating stability for refugee children, young people, and their families” [72] (p. 27). Stability is essential for any child to reach their full potential [73], and is a central tenet as to why Canada resettles refugees [74]. Moreover, schools are one of the key institutions that promote refugee integration [65], and thus the major disruptions caused by the pandemic have the potential to have a profound impact on the experience of refugees resettled in Canada.

### 3.8. Closure of Community and Religious Centres

Integration is one of Citizenship and Immigration Canada’s (CIC) core policy goals. The CIC’s commitment is demonstrated in the budgets released in the annual Report on Plans and Priorities. The 2015–2016 report budgeted over one billion dollars to Program 3.1: Newcomer Settlement and Integration, consequently receiving the most financial resources of any program described in the report [75]. The program aims to “strengthen social integration by providing newcomers with tools to fully participate in the labour market; promoting social and cultural connections; encouraging active civic participation; and fostering a sense of the rights and responsibilities of Canadian citizenship” [75]. The CIC financed programming is open to all newcomers to Canada who are permanent residents, including refugees, who have been in Canada for under 10 years. Measurement of integration is challenging, in part due to the lack of a control group to make comparisons against, but one of the performance indicators used to measure the success of Program 3.1 is the “percentage difference of newcomers who are 15 years and older, who in the past 12 months volunteered, or participated at least monthly in a group, association or organization, in comparison with the Canadian-born population” [75]. The target for this indicator is 10%. Social integration is clearly important to the Canadian government.

It is also important to refugees. Unlike other classes of immigrants, GARs tend to have few social ties in Canada, thereby lacking social capital upon their arrival [76]. Studies have found a bi-directional relationship between the strength of social connections and employment, education, and language skills [77]. Interpersonal relationships between community members and refugee newcomers are thus crucial for successful integration, benefiting refugees and fulfilling government aims alike. Despite being a high-level goal of the Canadian government, integration happens at the local level, with neighbourhood communities playing a major role in welcoming refugees and offering social support [76]. However, in mid-March 2020, public facilities across Canada were closed due to the COVID-19 pandemic [78]. As a result, social integration efforts via community channels such as community centres and places of worship have been greatly challenged.

While the IRCC does not collect data on the religion of refugees or asylum seekers [79], it is important to acknowledge the crucial role religion can play in refugee’s lives upon resettling in Canada. Refugees tend to come from countries where the experience of religion is common, and religion can offer its followers strength in challenging times, such as the pandemic in question [80]. It is also a platform through which refugees can integrate into their new community. Many places of worship have done their best to adapt to COVID-19 circumstances. For example, churches have live-streamed sermons on social media [81], and cities in Ontario, such as Mississauga and Toronto, have allowed mosques to broadcast the call to prayer over a loudspeaker, something usually prohibited under municipal codes [82]. These efforts have no doubt brought comfort to many during a challenging time.

Nonetheless, as platforms for social integration, physical distancing has potentially hampered integrative efforts in religious and community spaces. Of course, the opposite could be true; generally, community solidarity has been widespread in Canada amidst the pandemic, and it is thus possible that communities have made extra efforts to check in on their refugee neighbours. Further research is needed to understand how the closure of public spaces in relation to COVID-19 has impacted the social integration of refugees. Acknowledging the negative impact an extended period of physical distancing can have on mental health [83], and knowing refugees may already face mental health challenges due to past trauma [84], understanding how COVID-19 has impacted social supports is crucial.

### 3.9. Closure of Borders

“This virus does not respect borders,” repeated Tedros Adhanom Ghebreyesus, the Director-General of the WHO, on 27 February 2020 [85]. Due to the lack of scientific evidence proving the effectiveness of travel restrictions in most public health emergencies, the WHO has continued to advise against travel or trade restrictions during the COVID-19 pandemic. Instead, the WHO has promoted screening at points of entry, and the collection of traveller information to enable strong contact tracing as part of their recommendations for international travel [86]. Nonetheless, on 16 March 2020, Canada announced its intention to close international borders, extending the closure to its border with the United States on 18 March 2020 [64]. On 18 April 2020, the U.S.–Canada border closure was extended for a further thirty days [87]. Though many countries have done the same, these border closures are in violation of the International Health Regulations (2005) [88]. Border closures indicate a collective health security approach to managing the spread of the virus.

The border closures have been a cause for concern among refugee groups in Canada. Alex Neve, the secretary-general of Amnesty International Canada, has stated that:

“*Canada’s decision is out of step with public health measures designed to curb the spread of COVID-19, and runs counter to our international legal obligations. From moral, public health and legal perspectives, closing the border to refugee claimants is wrong. Turning refugee claimants away—including as a result of the decision to shut down the Canada/US border—exposes refugees, who face increased hardship, danger and ostracization worldwide related to this pandemic, to serious human rights violations, including inhumane immigration detention conditions and the risk of refoulement to torture and other human rights abuses*.”

[89]

Other refugee groups in Canada, including the Canadian Council for Refugees and the Canadian Association of Refugee Lawyers, have expressed viewpoints which echo Neve’s. One of their chief concerns is the conditions of the detainment facilities that asylum seekers attempting to enter Canada via the U.S.-Canada border will likely face upon return to the U.S. [90]. In the context of a pandemic, this is a cause for great concern given the high risk of being exposed to COVID-19 in crowded detention facilities [91].

Canada also houses asylum seekers in detention facilities, located in Laval, Quebec, Toronto, Ontario, and Surrey, British Columbia, [92] which have been similarly criticized as being unsafe during the COVID-19 pandemic [91]. In response, the Immigration and Refugee Board of Canada, the administrative tribunal responsible for decisions on refugee matters, has made strong efforts to release those held in provincial jails and immigration holding centres. The numbers indicate that over the course of one month (17 March–19 April 2020), the number of detainees dropped by 50%, an unprecedented action [93].

On 21 March 2020, Canada stopped allowing asylum seekers to enter the country at unauthorized points of entry [94]. Asylum seekers are those who have fled their country of origin and are asking for protection in another country (in this case Canada), though it is undetermined whether a claimant is a refugee or not until their case has been decided in court [95]. While the majority of refugee claims in Canada are made by those using regular channels, these unauthorized points of entry are still well-used. For instance, in 2018, 20,603 asylum seekers sought entry through irregular border crossings, 3307 of which were granted refugee status, and 13,470 of which are still pending [96]. The closure of these unauthorized points of entry during the pandemic is a further point of contention with refugee advocates. Janet Dench, the executive director of the Canadian Council for Refugees, argues that this will not stop asylum seekers from entering the country, instead saying that “it is pretty much inevitable that people will now move to alternative routes” [94]. These new routes may be more dangerous, as people will be attempting to cross where they expect they will not get caught [94]. This is evidenced by the increased rates of human smuggling seen since the Canada-U.S. Safe Third Country Agreement passed; people will still come, just in more dangerous ways [97]. With the clamp-down on unauthorized points of entry, these asylum seekers will no longer have access to the supports that were previously available to them upon their entry to Canada [94].

Additionally, the United Nations High Commissioner for Refugees and the International Organization for Migration have temporarily suspended resettlement movements for overseas refugees and so too has the IRCC [98]. Even the resettlement process for refugees who were privately sponsored but had not yet arrived in Canada has been suspended “until further notice” [99]. Already it takes up to four months for refugees to arrive in Canada after their private sponsorship application has been approved, with COVID-19 now extending this timeline indefinitely [100]. The suspension of refugee resettlement programs will have serious impacts on the individual lives of refugees who were destined to start afresh in Canada. Remember, unlike immigrants who choose whether or not to move to Canada, refugees have been forced to flee their country of origin because it was not safe for them [101]. With the recent halt to refugee programs, more people will be left in refugee camps and other temporary informal settlements. These environments often have their own health and safety risks. Refugee camps, for example, often have very limited health care capacities, are very densely populated, and fears of the devastation that COVID-19 could wreak in these camps are widespread [102]. The re-establishment of resettlement efforts is crucial to support refugee burden sharing.

## 4. Conclusions

As various health security measures are implemented across Canada, it is important to consider the unique lived experience of refugees during the COVID-19 pandemic. The challenges refugees encounter, as discussed in this article, include barriers to healthcare, economic support, education, social support, and border crossing impediments, all of which can have a compounding effect. This article has aimed to frame the issue and set the context; however, further research listening to the voice of the refugees in question is a crucial next step in the process to understand the experience of refugees in Canada. The refugee perspective is particularly needed to understand which barriers matter most to them, which will in turn help refugee support services best direct their resources. At this time much is unknown about how long COVID-19 will challenge the way Canadians live their lives. Whether for this pandemic or the next health security emergency, understanding how to best support the most vulnerable in Canadian society during an international crisis needs to be a high priority. Doing so will support efforts to develop effective prevention policies and strategies that will benefit not only refugees in Canada, but the entire population.

## Data Availability

The study did not report any data.
